# Factors influencing inpatient rehabilitation length of stay following revision hip replacements: a retrospective study

**DOI:** 10.1186/1471-2474-11-252

**Published:** 2010-10-28

**Authors:** So-Mei Teresa Yeung, Aileen M Davis, Rajka Soric

**Affiliations:** 1 Musculoskeletal Service, West Park Healthcare Centre, 82 Buttonwood Ave, Toronto, ON, M6M 2J5, Canada; 2 Division of Health Care and Outcomes Research, Toronto Western Research Institute, MP11-322, 399 Bathurst Street, Toronto, ON, M5T 2S8, Canada; 3 Faculty of Medicine, University of Toronto, Medical Sciences Building, Toronto, ON, M5S 1A8, Canada

## Abstract

**Background:**

The annual incidence of revision hip replacements has increased in both Canada and United States, particularly in younger adults. Patients following revision hip replacements often require longer hospital length of stay (LOS) but little is known about predictors of inpatient rehabilitation LOS in this group of patients. The purpose of this study was to evaluate the socio-demographic, pre-surgery, surgery and post-surgery related factors that might influence rehabilitation LOS of inpatients following revision hip replacements.

**Methods:**

This study included inpatients discharged from a musculoskeletal ward between 2002 and 2006 following rehabilitation revision hip replacement. Data sources included the National Reporting System, a standardized, provincial administrative database and augmented by chart abstraction. The collected elements included the outcome LOS and the following independent variables: age, sex, support at home, environmental barriers, language barrier, number of revision surgeries on the affected hip, comorbidity, previous orthopaedic surgeries in the lower extremities (L/ES), the hip component(s) revised, weight-bearing status, hemoglobin level, complications, days lapsed from surgery to rehabilitation admission and admission scores on the Functional Independence Measure (FIM). Simple linear regression was used to take forward any predictors significant at p < .10 level. Variables that satisfied the significance level were grouped in blocks and entered for regression analyses.

**Results:**

The 275 patients in this sample had a mean age of 69 years; 62% were female and the mean LOS was 29.6 days. Statistically significant predictors of longer LOS were low admission FIM score, female sex, revision of only the femoral component, 2 or more prior surgeries in the L/Es and 2 or more hip revisions (redo revision). The final model explained 28% of variance in inpatient LOS.

**Conclusions:**

A score of 9-14 points lower in admission FIM, female sex, revision of only the femoral component, prior surgeries in the L/Es and redo hip revision are all independent factors associated with 4-6 days longer LOS. These results may facilitate an understanding of bed flow. Additionally, patients with one or a combination of the above characteristics may benefit from enhanced care plans that facilitate achievement of rehabilitation goals for discharge home.

## Background

With the increase in annual incidence of total hip replacement (THR) in the past 2 decades [1-3], the number of revision hip replacements is expected to rise. Revision burden, which refers to the percentage of revision hip replacements relative to the total number of primary and revision hip replacements, ranged from 11% to 18% among different countries in the past 2 decades [4]. The revision burden stayed roughly the same in the United States (14-17% from 1993 to 2005) [1] and in Canada (11-13% from 2001 to 2006) [5,6]. This relatively constant rate reflected an increase in the absolute number of revisions as the number of primary procedures, particularly in younger adults, had increased [1,3]. Data from 1997-2005 indicated that the proportion of patients with THR in adults younger than 65 years old had increased from 26.5% to 43.7% in the United States [1]. In Canada, the largest 10-year increase in THR in males was seen among the 45-54 age group followed by the younger-than-45 age group. For females, the second and the third largest increases were seen respectively in the 55-64 and 45-54 age groups [3]. Unless arthritis related research such as cartilage regeneration effectively delay and/or avoid the need for THR, or important breakthroughs substantially increase the lifespan of hip prosthesis and/or the material for prosthetic fixation, the demands for primary THR and the subsequent revision hip replacements will continue to grow.

It is recognized that revision hip replacement is more complex and is associated with a higher complication rate than primary THR [7-9]. Patients following revision surgery often require longer stay in acute care [7,8,10] and subacute rehabilitation settings [11,12]. Patients following the more complex revision hip replacement are often discharged to subacute care setting for rehabilitation [1,8,9]. Yet, our knowledge and understanding of the factors that impact inpatient rehabilitation length of stay (LOS) after revision hip replacements are limited. Most studies on revision hip replacement were not in the area of LOS, but related to risk factors and indications for surgery or implant survival/surgical procedures,[13-18] function [7,12,19-21] or health related quality of life [22]. Studies on LOS on patients with revision surgery mostly focused on costs in the acute care settings [8,10,23], while a small number of studies evaluating factors that impact rehabilitation LOS for people with revision versus primary THR gave conflicting information. In one study, [12] the LOS was longer and the scores on Functional Independence Measure (FIM) was lower on admission and discharge in people with revision versus primary THR, but no difference in these 2 outcomes was observed between the 2 groups in another study [24].

The reason for revision hip replacement has been found to affect the rehabilitation outcomes between inpatients with initial and revision hip replacements. Inpatients who had their hip replacement revised for infection as opposed to mechanical loosening or pain had less improvement in FIM; they were also discharged home from rehabilitation setting less often when compared to inpatients with first hip replacement [12]. However, no studies have specifically considered factors that influence inpatient rehabilitation LOS in revision THR. The purpose of this study was to evaluate socio-demographic, pre-surgery, surgery and post surgery related factors that might influence rehabilitation LOS of inpatients following revision hip replacement. Identifying these predictors of LOS will facilitate improvements in care processes by informing care planning and more effective resource allocation. For example, more accurate prediction of LOS should improve access to inpatient beds and allow improved allocation of resources [25] such as staffing. These changes may ultimately translate into improved system efficiencies and patient outcomes. Considering the anticipated increase in revision hip procedures and the corresponding demands on rehabilitation inpatient beds, it is important that we gain a better understanding of the factors influencing rehabilitation LOS after revision hip replacements.

## Methods

### Sample

Participants in this study included all patients discharged between April 2002 and December 2006 from the Complex Musculoskeletal ward of a freestanding community rehabilitation centre in Toronto with an admission diagnosis of revision hip replacement. Potentially eligible participants were identified from an administrative database. Exclusion criteria included wound dehiscence, use of skin or muscle flap during surgery and first stage revision (i.e. removal of prosthesis and/or insertion of spacer). The study was approved by the Institutional Research Ethics Board.

### Setting

The inpatient ward where participants were treated accepted all referrals with musculoskeletal conditions but priority was given to people with diagnoses relating to revision joint replacement and/or multiple traumas. Additional admission criteria included ability to actively participate in therapy for at least an hour per day, identified realistic goals for rehabilitation and identified discharge destination. Functional discharge criterion was safe independent or supervised mobility in a physical environment similar to patient's planned discharge destination. The anticipated time for achieving this discharge criterion was discussed at the weekly team rounds in the first week of a patient's admission; a discharge date was determined accordingly. This discharge date was discussed among team members informally on a daily basis and readjusted as appropriate.

All newly admitted patients were assessed by the interdisciplinary rehabilitation team that includes the care-coordinator, nurses, pharmacist, attending physician, consultant physiatrist, occupational and physical therapists. No formal care path was used for patients with revision hip replacement at the time of this study but all admissions followed routine procedures. All patients were assessed and treated within 24 hours of admission and medically stable patients were assisted to move around as per their activity tolerance. Following the admission assessments, the team and the patient established mutually agreeable rehabilitation goals and the treatment plan for his/her inpatient stay. All patients participated in their individualized occupational and physiotherapy treatment sessions at least once a day for an hour or more on weekdays. Weekend occupational and physical therapies were provided to a selected group of patients who were identified by the case therapist as being at risk of pulmonary complications or deterioration in body function/structures and activity limitations. Patients were also instructed to perform exercises independently as appropriate for their conditions. Mobility training was incorporated into the nursing care plan and integrated into patients' activities of daily living (e.g. to assist/supervise patient to walk to the washroom). Therapy from other disciplines such as clinical psychologist, recreational or respiratory therapy was provided as needed.

### Data Collection

Data were abstracted from the National Reporting System (NRS). The data were augmented by chart abstraction by study investigator S Yeung and a trained research assistant using standardized operational instructions. Using the codes for revision hip replacement, all medical records of patients discharged between April 2002 and December 2006 with an admission diagnosis of revision hip replacement were identified from the NRS. The NRS is a standardized mandatory administrative database for all designated rehabilitation beds in Ontario [26]. For this study, the following data were extracted: date of birth, sex, spoken language, date of admission and discharge, admission diagnosis, comorbidity and admission and discharge scores of the FIM. Because only a limited list of comorbid conditions could be recorded on the menu of the NRS, data on comorbidity were supplemented by direct chart abstraction.

### Study Design and Measures

This was a retrospective study; the outcome was inpatient rehabilitation LOS in days. Predictors that might affect LOS were: socio-demographic related variables (age, sex, availability of support, environmental barriers at discharge destination and language barrier), pre-surgery related variables (number of revision surgeries in the hip, comorbidities and previous number of orthopaedic surgeries in the joints of lower extremities), surgical variable (the component being revised) and post-surgery related variables (weight bearing status on rehabilitation admission, hemoglobin level on rehabilitation admission, medical and/or orthopaedic complications, number of days from surgery to rehabilitation admission and admission scores on the FIM motor subscale).

Support was defined as available when a specific source (e.g. spouse, friends) was recorded as available when needed by patients during daytime and/or night time in the medical record. Presence of environmental barrier was defined by a record of presence of any stairs (≥2 steps) to essential living area at discharge destination. These included entrance to an apartment, bedroom, washroom, and kitchen etc., where no alternative access such as ramp or elevator is available. Presence of language barrier was defined by a record of spoken language other than English. The number of revision surgeries was defined as the total counts of surgeries related to hip revision that included removal of one or both components of hip prosthesis/spacer and/or implantation of a new implant. Therefore, a 2-stage revision was counted as 2 revisions to account for the clinical/physiological consequences of elective soft tissues trauma and the recovery which a patient had to encounter following each surgery. Comorbidity was recorded as the number of documented comorbid conditions included in the American Academy of Orthopaedic Self-report Comorbidity Questionnaire (AAOSC) [27]. Rheumatoid arthritis, osteoporosis and previous fracture were assigned to the three conditions originally provided as open-ended in AAOSC. These conditions were selected because they were frequently encountered in the rehabilitation ward of the current study. Previous number of orthopaedic surgeries in the lower extremities was defined as the total number of orthopaedic surgeries in both lower extremities excluding all hip replacement surgeries in the recently operated hip. A checklist of orthopaedic and medical complications encountered post-orthopaedic surgery [7,24,28-30] was used to record complications.

The FIM was completed within 72 hours of admission and again at discharge by certified assessors of the rehabilitation team. Only the motor subscale of the FIM (motor FIM) was used in this study because admitted patients, in general, had no cognitive problems. This 13-item motor FIM assesses patient's performance on basic activities of daily living [31,32]. Scoring of each item ranges from 1 (total dependence) to 7 (complete independence) based on a standardized set of criteria. Scores on the motor FIM range from 13-91; lower score indicates lower function and more dependence in basic functional activities. The FIM was found to be reliable [33], valid and able to detect changes in a variety of inpatients including those with orthopaedic or traumatic conditions [34-36].

### Analyses

All analyses were conducted using the Statistical Package for the Social Sciences version 16.0. Descriptive statistics including means, standard deviation or frequencies were calculated for all variables. Variables that were treated as continuous were age, admission hemoglobin, number of days post-surgery on admission to the inpatient ward and admission FIM scores. Variables that were treated as non-continuous were dichotomized based on clinical judgment of the impact of these variables on inpatient LOS and/or the distribution of data that allowed adequate information for analyses. For example, the number of revision surgeries was dichotomized as 1 and ≥2 based on clinical observations that patients who had their hip revised more than once often require longer time to regain independence in basic functional mobility when compared to patients following the first revision. Previous number of orthopaedic surgeries in the lower extremities, and the number of comorbid conditions were both dichotomized as ≤1 and ≥2. Admission weight bearing status was dichotomized as no/touch weight bearing and partial/as tolerated weight bearing. The 4 variables on complication (medical or orthopaedic), environmental barrier, language barrier and availability of support were all dichotomized as absence and presence. The variable on "component being revised" was categorized as "only the acetabular component", "only the femoral component" and "both acetabular and femoral components"; two dummy variables were created for this variable for the regression analyses described below. The category of "only the acetabular component" had the shortest length of stay and was selected as the reference category to compare with the other 2 categories.

After evaluating the assumptions for linear regression, regression was used to analyze the relationship of LOS and all predictor variables. Simple linear regression was used to take forward any predictors that were significant at the p < .10 level. Variables that satisfied the significance level were selected and grouped in blocks of socio-demographic, pre-surgery, surgery and post-surgery related characteristics. These blocks were then manually entered for regression analyses in 4 steps. In the first step, only the socio-demographic block was entered, and the p-values of the variables in this block were manually assessed for inclusion (p < .10) in the next step. In the second step, only the socio-demographic and the pre-surgery blocks were entered. The surgery block was added in the third step and the post-surgery block was added in the fourth step. The manual assessment was repeated for all variables used in each step after an additional block was entered to determine inclusion of variables used within each block in the subsequent steps. The 2 dummy variables, "only the femoral component" and "both acetabular and femoral components" were force entered together in the final step of the model building.

## Results

Two hundred and ninety seven patients with revision hip replacement were identified within the stated study period; 22 cases matched the exclusion criteria and were excluded, leaving a total of 275 cases for analyses. Missing data ranged from 0.4% to 3%. The exception was "component being revised" in which 37/275 or 13.5% of participants had missing data. There were no significant differences in the samples with and without missing data. Results of the sample producing the regression model are presented below.

The mean age of the sample was 69.1 years old; 62% were female. The LOS ranged from 4 to 91 days with a mean of 29.6 days (s = 16.4). Forty-four percent of patients were admitted for revision of a previously revised hip, referred to as redo revision from hereon. Majority of patients (89.8%) were discharged home; 2.9% were discharged to acute care for orthopaedic or medical reasons (e.g. fractures, hip dislocation, respiratory failure, heart attack etc.) and 6.9% were discharged to supported living settings. The mean score for the motor FIM on admission was 47.6. The socio-demographic and pre-surgery related characteristics of the sample are summarized in Table 1. The surgery and post-surgery related characteristics, and the LOS of the sample are shown in Table 2.

**Table 1:** Socio-demographic and Pre-Surgery related characteristics (N = 275)

	n	%*	Mean, SD, Min-Max
Demographics			
Age, yrs			69.1, 11.9, 22-87
Female sex	170	61.8	
Have support available at home	176	64.0	
Have environmental barriers at home	139	50.5	
Have language barrier	28	10.2	
Pre-surgery related variables			
Number of hip revisions^†^			1.8, 1.2, 1-6
1^st^	155	56.4	
Redo revisions (≥2)	120	43.6	
2^nd^	66	24.0	
3^rd^	23	8.4	
4^th^-6^th^	31	11.3	
Number of previous L/Es surgeries^‡^			1.5, 1.5, 0-10
≤ 1	169	61.5	
≥2	105	38.3	
0	78	28.4	
1	91	33.1	
2	51	18.5	
3-4	44	16.0	
5-10	10	3.7	
Number of comorbid conditions			2.0, 1.4, 0-6
≤1	105	38.2	
≥2	164	59.6	

* Percentage may not add up to 100 related to missing data;

†In the hip responsible for the current rehabilitation admission

‡ Total number of orthopaedic surgeries in the lower extremities excluding all hip replacement surgeries in the recently operated hip.

L/Es, lower extremities

**Table 2:** Surgery and Post-surgery related characteristics (N = 275)

	n	%*	Mean, SD, Min-Max
Revised component			
Acetabular only	77	28.0	
Femoral only	60	21.8	
Acetabular & Femoral	101	36.7	
Weight bearing restriction on admission			
NWB or TWB	189	68.7	
NWB	150	54.5	
TWB	39	14.2	
PWB or WBAT	85	28.7	
PWB	19	6.9	
WBAT	66	24.0	
No. of patients with medical and/or orthopaedic complications	119	43.3	
No. of medical complication			
0	185	68.8	
1-2	75	27.1	
3-4	9	3.3	
No. of orthopaedic complication			
0	218	81.0	
1	46	16.7	
2-3	5	1.8	
No. of days after surgery on admission			8.7, 8.0, 3-63
3-4	63	22.9	
5-7	107	38.9	
8-14	78	28.4	
≥15	27	9.8	
Hemoglobin on admission (gram/liter)			98.0, 12.1, 6-13
Length of Stay^†^			29.6, 16.4, 4-91
Motor FIM on admission			47.6, 11.7, 13-81
Motor FIM on discharge			75.2, 9.4, 20-84

*Percentage may not add up to 100 related to missing data;

† Median = 27 days, Q1 = 17 days, Q3 = 40 days, mode = 21 days

NWB, no weight bearing; TWB, touch weight bearing; PWB, partial weight bearing; WBAT, weight bearing as tolerated

The unadjusted relationships between LOS and all potential predictors are summarized in Table 3. The variables on environmental barriers and language barrier did not satisfy the .10 level of significance in their corresponding simple linear regression with LOS and were excluded in further models. The results in each step of the regression are shown in Table 4 and the final model is shown in Table 5. Factors that were significant (p < .05) in predicting longer LOS were low admission FIM score, female sex, "only the femoral" as compared to "only the acetabular" revision, had 2 or more prior surgeries in lower extremities and redo revision (≥2 revisions). These 5 factors accounted for 28.0% of variance in the inpatient rehabilitation LOS. The independent effect of all 4 dichotomous variables on LOS ranged from 4-6 days. A similar impact in longer LOS was observed with a score reduction of 9-14 points in admission FIM (Table 5).

**Table 3:** Unadjusted relationship between Length of Stay (LOS) in days & all potential predictors

Continuous Variable		N	LOS		r	p-value
					
			Mean	SD		
Age		275	30	16.4	.189	.002
Adm. Hemoglobin (g/l)		267	30	16.2	.108	.079
Days after THR on admission		275	30	16.4	.149	.013
Motor FIM on admission		261	30	16.7	-.440	<.001

Dichotomized Variables						

Female sex	No	105	26	16.0		.001
	Yes	170	32	16.1		
Had support	No	95	33	18.0		.005
	Yes	176	27	15.4		
Environmental Barriers	No	128	31	16.8		.142
	Yes	139	28	16.2		
Language Barrier	No	246	30	16.7		.844
	Yes	28	29	13.9		
Redo revision (≥2)	No	155	27	13.9		<.001
	Yes	120	34	18.3		
Had 2 or more prior L/Es surgeries	No	169	28	15.3		.026
	Yes	105	32	17.7		
Had complication(s)	No	150	27	15.0		.011
	Yes	119	32	17.7		
	
		**N**	**LOS**			p-value*
					
			**Median**	**IQR**		

Had 2 or more comorbid conditions	No	105	23	17.5		.004
	Yes	164	30	24.0		
Revised component						
Acetabular only	No	161	29	22.0		.001
	Yes	77	21	15.5		
Femoral only	No	178	26	19.0		.026
	Yes	60	33	24.8		
Acetabular & Femoral	No	137	23	20.5		.186
	Yes	101	29	19.0		
P/WBAT on admission	No	189	30	24.0		<.001
	Yes	85	21	15.5		

*Significance of Non-parametric test

r, Pearson correlation; Adm., admission; THR, total Hip replacement; FIM, Functional Independence Measures; L/Es, lower extremities; P/WBAT; partial or weight bearing as tolerated

**Table 4:** Results of the regression analyses (Dependent variable was length of stay in days)

		Step 1		Step 2*		Step 3*		Step 4*	
		B1 only		B1 & 2		B1, 2 & 3		B1, 2, 3 & 4	
Block (B)	Variable								
		Adjusted β, p		Adjusted β, p		Adjusted β, p		Adjusted β, p	
1. Socio-demographic variables	Age	.20,	.001	.14,	.022	.17,	.006	.08,	.225
	Female	.20,	.001	.17,	.005	.24,	<.001	.19,	.003
	Had Support	-.12,	.044	-.15,	.010	-.04,	.489		

2. Pre-surgery related variables	Redo revision (≥2)			.20,	.001	.11,	.085	.12,	.048
	Had 2 or more prior L/Es surgeries			.10,	.076	.16,	.008	.14,	.027
	Had one or more comorbid conditions			.10,	.106				

3. Surgery related variables	Acetabular only revision					.00		.00	
	Acetabular & femoral only revision					.22,	.003	.11,	.143
	Femoral only revision					.28,	<.001	.17,	.025

4. Post-surgery related variables	P/WBAT on admission							-.12,	.058
	Admission Hemoglobin							-.01,	.871
	Had complication(s)							.02,	.712
	Days after THR on admission							.07,	.316
	Admission motor FIM							-.31,	<.001

*Only variables significant at p < .10 at the previous step were retained and entered in the step.

L/Es. lower extremities; P/WBAT, partial or weight bearing as tolerated; THR, total hip replacement; FIM, Functional Independence Measures

**Table 5:** Final model* of the regression analyses (n = 212, dependent variable was length of stay in days)

Model	Unstandardized Beta	Std. Error	Standardized Beta	p-value
(Constant)	40.27	11.54		.004
Female	6.30	2.09	0.19	.003
Redo revision (≥2)	4.00	2.01	0.12	.048
Had 2 or more prior L/Es surgeries	4.55	2.04	0.14	.027
Acetabular only revision	0.00			
Acetabular & Femoral revision	3.53	2.40	0.11	.143
Femoral only revision	6.43	2.84	0.17	.025
Admission FIM scores	-0.44	0.09	-0.31	<.001
Age	0.11	0.09	0.08	.225
P/WBAT on admission	-4.36	2.28	-0.12	.058
Admission Hemoglobin	-0.01	0.09	-0.01	.871
Had complication(s)	0.78	2.11	0.02	.712
Days after THR on admission	0.15	0.15	0.07	.316

*Total adjusted R^2 ^= 0.28

L/Es, lower extremities; P/WBAT, partial or weight bearing as tolerated, THR, total hip replacement

## Discussion

The rising incidence of primary and revision hip replacements in the past few years [1,3,6] will likely increase demands for access to inpatient rehabilitation. Identification of factors that impact rehabilitation inpatient LOS will allow better prediction of bed flow and access, support effective resources allocation and potentially inform better care planning. To the authors' knowledge, this is the first study aiming to identify predictors of rehabilitation LOS specific to inpatients following revision hip replacement. The inpatient LOS for this sample was 29.6 days. Our results demonstrated that low admission FIM score, female sex, "only the femoral" as compared to "only the acetabular" revision, 2 or more previous surgeries in the lower extremities and redo revision predicted longer LOS.

Our finding that admission FIM score predicted inpatient LOS is not surprising. A common goal for most inpatients undergoing rehabilitation is to achieve the functional independence needed to return home. Patients who scored low on the FIM on admission were more dependent in basic functional activities when compared to patients who had higher scores, and may accordingly take longer to achieve safe and independent/supervised mobility needed to return home. Admission FIM score has been found to associate with longer LOS on inpatients with hip fractures [37] and stroke [38], but whether FIM can predict LOS has not been previously examined in people following joint replacement. Our current understanding is that when there were differences in both LOS and admission FIM scores between two comparison groups with THR, the group with lower admission FIM score had longer LOS [12]. Incidentally, when there was no difference in admission FIM scores between groups, there was also no difference in LOS between groups [39].

Our study confirms that basic functional independence as reflected by the FIM score is an important area in inpatient rehabilitation. While there is no doubt that basic functional training is the focus of most rehabilitation programs, a special care plan for training functional independence in patients who may have longer LOS may further improve this outcome. Further research is needed to examine if different intervention strategies (e.g. altering the intensity, frequency and/or duration of functional training) in patients with low admission FIM score will shorten the rehabilitation LOS of inpatients following revision hip replacement. It is also important to note that low FIM score implies more caregiver burden (i.e. more demands on resources). With the pressure of cost-containment, it remains unknown whether these additional demands have become or will become a subtle barrier for the more functionally dependent patients to have equal and timely access to inpatient rehabilitation. This area deserves further study to ensure that the rehabilitation needs of the low functioning patients are appropriately met.

Female sex was found to predict longer LOS in this study. This finding was consistent with data in patients with mixed cases of primary and revision hip replacement, where women were found to stay 1-2 days longer in both the acute care and in inpatient rehabilitation settings [6,40]. However, neither the independent effect of female sex on LOS nor data on patient with only revision hip replacements were reported [6,40] for further comparison. The magnitude of the independent effect of female sex in predicting 6 days longer LOS in this study is quite large. This result indicates that substantially slower bed flow and higher cost of rehabilitation should be expected in inpatient settings with more female patients who underwent revision hip replacements.

This study demonstrated that revision of only the femoral component predicted 6 days longer LOS (Table 5) when compared to revision of only the acetabular component. The deep and bow-shaped femoral canal has made it challenging to completely remove the cement mantle, or to retrieve the old ingrown femoral prosthesis and to prepare femoral canal for revision [41-43]. Controlling damages in bone and soft tissues, and avoiding devascularisation of the bone may be more difficult in femoral than acetabular revision. However, it is unclear why revision of both hip components (femoral and acetabular) did not predict longer LOS. It may be that other surgery related factors (e.g. bone graft) varies widely in this subgroup of patients, making it difficult to achieve a significant effect in LOS. It is recognized that a major challenge in any type of hip revision is bone deficiency [44], and the severity of which depends on the indication and timing of the revision. Including these 2 factors and information on bone graft in future studies may help to give better insight on the impact of revised components on LOS.

Prior surgeries (≥2) in the lower extremities predicted longer inpatient LOS in this sample of patients. We could not find similar data in samples with only revision hip replacement for comparison. Residual body impairment from prior surgeries may further worsen activity limitation, and a longer time may be needed to achieve basic functional independence. For example, a patient who has only 70 degrees of knee flexion on the contralateral side may take longer time to master the body mechanics needed to stand up from a chair while keeping the hip flexion less than 90 degrees on the operated side (standard hip precaution). In addition to the lower extremities, impairment of the upper extremities may also affect mobility because most inpatients rely on a walking aid, such as a walker, to move around. Unfortunately data on the impairment of upper extremities were not documented in a standardized manner and was not feasible to be used for quantitative analyses. Future prospective studies should consider the impact of all 4 extremities to obtain a more comprehensive picture of their effect on inpatient LOS.

Our results indicated that redo revisions are associated with 4 days longer LOS when compared to the first revision. It is presumably more challenging to restore normal anatomy and biomechanics of the hip with each additional revision. Some body impairments may remain after each revision surgery and accumulate to a 'threshold' to affect the pace of restoring basic functional independence. Limited data are available on the effect of revision number on LOS on inpatients following hip revisions. In their study comparing patients with primary and revision THR, Walker et al [24] found no difference in LOS between patients with first revision (n = 30) and redo revision (n = 9); details on revision number within the later group are not available. When compared to their sample, our sample had a larger number and proportion of patients with redo revision (120 versus 9 and 43.6% versus 23%, respectively). We also had a heterogeneous sample where patients' number of revision ranged from 2-6 in the subgroup with redo revision. These differences in sample size and patient characteristics may have contributed to the differences between our findings and that of Walker et al [24].

The authors are unaware of any Canadian data on inpatients rehabilitation LOS following hip revision. The average inpatient LOS observed in this study was longer than data from the United States (U.S.), which ranged from 10 to 15 days [11,12,24,45]. Differences in patients' characteristics or discharge criteria might have contributed to difference in LOS among different studies. In a small sample of 39 inpatients of average age 74.6 years, Walker et al [24] found that the rehabilitation LOS was 10.5 days. Although the sample of Walker et al [24] had comparatively more female patients than our sample (80% versus 62%), their sample had a lower proportion of patients with redo revision (23% versus 43.6%) and higher proportion of patients with revision of only the acetabular component (46% versus 28%). In another study where a subsample of 147 inpatients of average age 74 years old with revision hip replacement, Vincent et al [12] reported a rehabilitation LOS of 11.5 days. However, the sample of Vincent et al [12] also had lower discharge motor FIM score (54-65 for various reasons of revision versus 75), and had lower proportion of patients returning home (83% versus 90%) when compared to our sample. Unfortunately, no data were available in the study of Vincent et al [12] on discharge criteria or subsample patient characteristics for further comparison. There were 3 other studies [11,40,45] with mixed samples of both primary and revision hip replacements; the reported LOS of the subsamples with revision hip replacement ranged from 11 to 15 days [11,40,45]. It is difficult to compare our finding with these studies because there were no baseline characteristics for the subsamples of patients with hip revisions; generalizing their characteristics from the full sample where most patients had primary THR will be subjected to bias. Difference in source of rehabilitation funding may also contribute to difference in LOS between samples from U.S. and Canada, but this notion will demand separate and detailed analyses of the funding system in the two countries and will not be discussed here.

Prior reports [11,40,45] on LOS in people with revision THR often generalized all revision cases in one group and were unable to provide information on potential difference in impact of redo revision from first revision. Where the impact of redo revision was studied [24], the sample size of this subgroup (n = 9) was small. This subgroup constituted only less than a quarter of the full sample and the range in number of revision is not provided to allow further comparison between studies. A strength of this study is that we had a decent number of patients in both subgroups of redo and first revision with similar group size (n = 120 versus 155), and there was a range in number of revisions from 2 to 6 in the redo revision subgroup to provide reference data for comparison with future studies. However, this study also has several limitations. Firstly, no care path was used at the time of this study which might have affected the LOS, but a recognized formal care path was not available in Ontario for revision hip replacements at the time of this study. Our setting had implemented routine care procedures and discharge plans shortly after patient's admission, and we believe patients were discharged as soon as they were ready. Secondly, inpatients' LOS is likely affected by many patient and non-patient related factors (e.g. availability of rehabilitation support in community). Inherent to the retrospective design of this study, not all variables of interest were available and recorded in a standardized manner appropriate for quantitative analyses. The current study did not examine the impact on LOS from many other factors of interest such as body mass index, socio-economic status, race, number of painful joints in the body, indications of revision and rehabilitation staff-patient ratio etc. Considering that many multi-dimensional factors can possibly affect LOS, more than a quarter of the variance in LOS (28.0%) were explained by our model, which likely reflected the depth of influence from factors selected for use in this study. Another limitation of this study is that data were collected from only one inpatient setting which limits the generalization of the result. Although there was no geographic limitation on applicants' region of residence, most patients lived nearby and were operated by the limited number of surgeons from the acute care hospitals in downtown Toronto. It remains unknown if the surgical practices and post-surgical protocols of these surgeons on THR are different from surgeons in other geographic areas. Further study is needed in samples from different geographic location to validate the factors that had influenced LOS in this study. Overall, this exploratory study has provided valuable information for future prospective study to better understand factors that impact inpatient LOS in people following revision hip replacement.

The predictors found in this study can facilitate our understanding in the potential LOS of rehabilitation inpatients. For example, with all other factors being equal, a difference of a motor FIM admission score of 40 versus 54 would predict that the individual with the lower FIM score would have a 6 days longer LOS. Alternatively, our work would predict that a female as compared to a male would have a 6 days longer LOS given equality of other factors.

## Conclusions

A score of 9-14 points lower in admission FIM, female sex, revision of only the femoral component, 2 or more previous surgeries in the lower extremities and redo revision are all independently associated with 4-6 days longer LOS. These results may facilitate bed flow. Additionally, patients with one or a combination of the above characteristics that prolong LOS may benefit from enhanced care plans that facilitate achievement of rehabilitation goals for discharge home.

## Competing interests

The authors declare that they have no competing interests.

## Authors' contributions

All authors participated in the conceptualization and the design of the study. SY coordinated the study, participated in data collection, performed all statistical analyses, interpreted the data, drafted and revised the manuscript. AMD provided assistance in data analyses and interpretation. Both AMD and RS helped to draft and revise the manuscript. All authors read and approved the final manuscript.

## Authors' Information

SY: Physical Therapist, Clinical Practice Leader, Musculoskeletal Service, West Park Healthcare Centre, Toronto, Canada; Clinical Lecturer, Department of Physical Therapy, Faculty of Medicine, University of Toronto, Canada.

AMD: Senior Scientist, Division of Population Health and Outcomes Research, Toronto Western Research Institute; Professor, Departments of Physical Therapy and Surgery and Graduate Departments of Health Policy, Management and Evaluation, Rehabilitation Science and the Institute of Medical Science, Faculty of Medicine, University of Toronto, Canada.

RS: M.D., F.R.C.P.C.; Physiatrist, Musculoskeletal Service, West Park Healthcare Centre, Toronto, Canada; Assistant Professor, Division of Physiatry, Faculty of Medicine, University of Toronto, Canada.

## Pre-publication history

The pre-publication history for this paper can be accessed here:

http://www.biomedcentral.com/1471-2474/11/252/prepub

## References

[B1] Tian W DeJoing G Brown M Hsieh C Zamfirov ZP Horn SD Looking upstream: Factors shaping the demand for postacute joint replacement rehabilitation Arch Phys Med Rehabil 2009 90 8 1260 8 10.1016/j.apmr.2008.10.035 19651260

[B2] Malchau H Herberts P Eisler T Garellick G Soderman P The Swedish Total Hip Replacement Register J Bone Joint Surg Am 2002 84-A Suppl 2 2 20 12479335 10.2106/00004623-200200002-00002

[B3] CIHI Canadian Institute for health information, hip and knee replacements in Canada-Canadian Joint Replacement Registry (CJRR) 2008-2009 Annual Report 2009

[B4] Kurtz S Mowat F Ong K Chan N Lau E Halpern M Prevalence of primary and revision total hip and knee arthroplasty in the United States from 1990 through 2002 J Bone Joint Surg Am 2005 87 7 1487 1497 10.2106/JBJS.D.02441 15995115

[B5] CIHI Canadian Institute for health information: Canadian Joint Replacement Registry (CJRR) Analytic Bulletin June 2004 2004

[B6] CIHI Hip and Knee Replacements in Canada: Canadian Joint Replacement Registry (CJRR) 2007 Annual Report 2008

[B7] Saleh KJ Celebrezze M Kassim R Dykes DC Gioe TJ Callaghan JJ Salvati EA Functional outcome after revision hip arthroplasty: a metaanalysis Clin Orthop Relat Res 2003 416 254 264 10.1097/01.blo.0000093006.90435.43 14646768

[B8] Bozic KJ Katz P Cisternas M Ono L Ries MD Showstack J Hospital resource utilization for primary and revision total hip arthroplasty J Bone Joint Surg Am 2005 87 3 570 576 10.2106/JBJS.D.02121 15741624

[B9] Zhan C Kaczmarek R Loyo-Berrios N Sangl J Bright RA Incidence and short-term outcomes of primary and revision hip replacement in the United States J Bone Joint Surg Am 2007 89 3 526 533 10.2106/JBJS.F.00952 17332101

[B10] Lavernia CJ Drakeford MK Tsao AK Gittelsohn A Krackow KA Hungerford DS Revision and primary hip and knee arthroplasty. A cost analysis Clin Orthop Relat Res 1995 311 136 141 7634568

[B11] Lin JJ Kaplan RJ Multivariate analysis of the factors affecting duration of acute inpatient rehabilitation after hip and knee arthroplasty Am J Phys Med Rehabil 2004 83 5 344 352 10.1097/01.PHM.0000124446.59395.16 15100623

[B12] Vincent KR Vincent HK Lee LW Weng J Alfano AP Outcomes after inpatient rehabilitation of primary and revision total hip arthroplasty Arch Phys Med Rehabil 2006 87 8 1026 1032 10.1016/j.apmr.2006.04.015 16876546

[B13] Springer BD Fehring TK Griffin WL Odum SM Masonis JL Why revision total hip arthroplasty fails Clin Orthop Relat Res 2009 467 1 166 173 10.1007/s11999-008-0566-z 18975043 PMC2600991

[B14] Best JT Revision total hip and total knee arthroplasty Orthop Nurs 2005 24 3 174 179 10.1097/00006416-200505000-00003 15928524

[B15] Gross AE Blackley H Wong P Saleh K Woodgate I The role of allografts in revision arthroplasty of the hip Instr Course Lect 2002 51 103 113 12064094

[B16] Gross AE Saleh KJ Wong P Acetabular revision using grafts and cages Am J Orthop 2002 31 4 213 215 12008852

[B17] Saleh KJ Kassim R Gross AE Bone assessment and reconstruction in revision hip surgery Am J Orthop 2002 31 4 183 185 12008846

[B18] Schinsky MF Della Valle CJ Sporer SM Paprosky WG Perioperative testing for joint infection in patients undergoing revision total hip arthroplasty J Bone Joint Surg Am 2008 90 9 1869 1875 10.2106/JBJS.G.01255 18762646

[B19] Davis AM Agnidis Z Badley E Kiss A Waddell JP Gross AE Predictors of functional outcome two years following revision hip arthroplasty J Bone Joint Surg Am 2006 88 4 685 691 10.2106/JBJS.E.00150 16595456

[B20] Davis AM Agnidis Z Badley E Davey JR Gafni A Gollish J Mahomed NN Saleh KJ Schemitsch EH Szalai JP Waddell JP Gross AE Waiting for hip revision surgery: the impact on patient disability Can J Surg 2008 51 2 92 96 18377748 PMC2386340

[B21] Jones CA Beaupre LA Johnston DW Suarez-Almazor ME Total joint arthroplasties: current concepts of patient outcomes after surgery Rheum Dis Clin North Am 2007 33 1 71 86 10.1016/j.rdc.2006.12.008 17367693

[B22] Ethgen O Bruyere O Richy F Dardennes C Reginster JY Health-related quality of life in total hip and total knee arthroplasty. A qualitative and systematic review of the literature J Bone Joint Surg Am 2004 86-A 5 963 974 15118039 10.2106/00004623-200405000-00012

[B23] Crowe JF Sculco TP Kahn B Revision total hip arthroplasty: hospital cost and reimbursement analysis Clin Orthop Relat Res 2003 413 175 182 10.1097/01.blo.0000072469.32680.b6 12897608

[B24] Walker WC Keyser-Marcus LA Cifu DX Chaudhri M Inpatient interdisciplinary rehabilitation after total hip arthroplasty surgery: a comparison of revision and primary total hip arthroplasty Arch Phys Med Rehabil 2001 82 1 129 133 10.1053/apmr.2001.18604 11239299

[B25] Capuano T Mackenzie R Pintar K Halkins D Nester B Complex adaptive strategy to produce capacity-driven financial improvement J Healthc Manag 2009 54 5 307 319 19831116

[B26] Rehabilitation (NRS) program at Canadian Institute of Health Research Hospital Report e-Scorecard 2008: Rehabilitation Clinical Utilization and Outcomes Technical Summary 2009 1 11

[B27] Sangha O Stucki G Liant MH Fossel AH Katz JN The self-Administered Comorbidity Questionnaire: A new method to assess comorbidity for clinical and health services research Arthritis Rheum 2003 49 2 156 163 10.1002/art.10993 12687505

[B28] DeJong G Tian W Smout RJ Horn SD Putman K Smith P Gassaway J Davanzo JE Use of rehabilitation and other health care services by patients with joint replacement after discharge from skilled nursing and inpatient rehabilitation facilities Arch Phys Med Rehabil 2009 90 8 1297 1305 10.1016/j.apmr.2008.12.029 19651263

[B29] Yasunaga H Tsuchiya K Matsuyama Y Ohe K Analysis of factors affecting operating time, postoperative complications, and length of stay for total knee arthroplasty: nationwide web-based survey J Orthop Sci 2009 14 1 10 16 10.1007/s00776-008-1294-7 19214682

[B30] Vincent HK Vincent KR Influence of Admission Hematocrit on Inpatient Rehabilitation Outcomes After Total Knee and Hip Arthroplasty Am J Phys Med Rehabil 2007 86 10 806 817 10.1097/PHM.0b013e318151fe19 17885313

[B31] Stineman MG Ross RN Fiedler R Granger CV Maislin G Functional independence staging: conceptual foundation, face validity, and empirical derivation Arch Phys Med Rehabil 2003 84 1 29 37 10.1053/apmr.2003.50061 12589617

[B32] Stineman MG Shea JA Jette A Tassoni CJ Ottenbacher KJ Fiedler R Granger CV The Functional Independence Measure: tests of scaling assumptions, structure, and reliability across 20 diverse impairment categories Arch Phys Med Rehabil 1996 77 11 1101 1108 10.1016/S0003-9993(96)90130-6 8931518

[B33] Ottenbacher KJ Hsu Y Granger CV Fiedler RC The reliability of the functional independence measure: a quantitative review Arch Phys Med Rehabil 1996 77 12 1226 1232 10.1016/S0003-9993(96)90184-7 8976303

[B34] Aitken DM Bohannon RW Functional independence measure versus short form-36: relative responsiveness and validity Int J Rehabil Res 2001 24 1 65 68 10.1097/00004356-200103000-00009 11302466

[B35] Czyrny JJ Kelley D Brentjans M Functional outcomes of patients with multiple limb trauma Am J Phys Med Rehabil 1998 77 5 407 411 10.1097/00002060-199809000-00008 9798832

[B36] Grimby G Gudjonsson G Rodhe M Sunnerhagen KS Sundh V Ostensson ML The functional independence measure in Sweden: experience for outcome measurement in rehabilitation medicine Scand J Rehabil Med 1996 28 2 51 62 8815989

[B37] Nguyen-Oghalai TU Ottenbacher KJ Granger CV Smith ST Goodwin JS Impact of osteoarthritis on rehabilitation for persons with hip fracture Arthritis & Rheum 2006 55 6 920 924 10.1002/art.22345 17139638

[B38] Tan WS Heng BH Chua KS Chan KF Factors predicting inpatient rehabilitation length of stay of acute stroke patients in Singapore Arch Phys Med Rehabil 2009 90 7 1202 1207 10.1016/j.apmr.2009.01.027 19577034

[B39] Nguyen-Oghalai TU Ottenbacher KJ Caban M Granger CV Grecula M Goodwin JS The impact of rheumatoid arthritis on rehabilitation outcomes after lower extremity arthroplasty J Clin Rheumatol 2007 13 5 247 250 10.1097/RHU.0b013e3181570ad4 17921790

[B40] Vincent HK Alfano AP Lee L Vincent KR Sex and age effects on outcomes of total hip arthroplasty after inpatient rehabilitation Arch Phys Med Rehabil 2006 87 4 461 467 10.1016/j.apmr.2006.01.002 16571383

[B41] Masterson EL Masri BA Duncan CP Surgical approaches in revision hip replacement J Am Acad Orthop Surg 1998 6 2 84 92 9682070 10.5435/00124635-199803000-00002

[B42] Choplin RH Henley CN Edds EM Capello W Rankin JL Buckwalter KA Total hip arthroplasty in patients with bone deficiency of the acetabulum Radiographics 2008 28 3 771 786 10.1148/rg.283075085 18480483

[B43] Taylor RH Joskowicz L Williamson B Gueziec A Kalvin A Kazanzides P Van Vorhis R Yao J Kumar R Bzostek A Sahay A Borner M Lahmer A Computer-integrated revision total hip replacement surgery: concept and preliminary results Med Image Anal 1999 3 3 301 319 10.1016/S1361-8415(99)80026-7 10710298

[B44] Hassaballa M Mehendale S Poniatowski S Kalantzis G Smith E Learmonth ID Subsidence of the stem after impaction bone grafting for revision hip replacement using irradiated bone J Bone Joint Surg Br 2009 91 1 37 43 10.1302/0301-620X.91B1.20376 19092002

[B45] Dejong G Horn SD Smout RJ Tian W Putman K Gassaway J Joint replacement rehabilitation outcomes on discharge from skilled nursing facilities and inpatient rehabilitation facilities Arch Phys Med Rehabil 2009 90 8 1284 1296 10.1016/j.apmr.2009.02.009 19651262

